# Autonomic nervous system dysfunction predicts poor prognosis in patients with mild to moderate tetanus

**DOI:** 10.1186/1471-2377-5-2

**Published:** 2005-01-31

**Authors:** Mohammad Wasay, Bhojo A Khealani, Naasha Talati, Rohmah Shamsi, Nadir A Syed, Naseem Salahuddin

**Affiliations:** 1 Department of Neurology, The Aga Khan University, Karachi, Pakistan; 2 Department of Medicine, The Aga Khan University, Karachi, Pakistan; 3 Department of Medicine, Liaquat National Hospital, Karachi, Pakistan

## Abstract

**Background:**

Autonomic nervous system (ANS) dysfunction is present in up to one third of patients with tetanus. The prognostic value of ANS dysfunction is known in severe tetanus but its value is not well established in mild to moderate tetanus.

**Methods:**

Medical records of all patients admitted with tetanus at two academic tertiary care centers in Karachi, Pakistan were reviewed. The demographic, clinical and laboratory data was recorded and analyzed. ANS dysfunction was defined as presence of labile or persistent hypertension or hypotension and sinus tachycardia, tachyarrythmia or bradycardia on EKG. Patients were divided into two groups based on presence of ANS dysfunction (ANS group and non ANS group). Tetanus severity was classified on the basis of Ablett criteria.

**Results:**

Ninety six (64 males; 32 females) patients were admitted with the diagnosis over a period of 10 years. ANS group had 31 (32%) patients while non ANS group comprised of 65 (68%) patients. Both groups matched for age, gender, symptom severity, use of tetanus immunoglobulin and antibiotics. Twelve patients in ANS group had mild to moderate tetanus (Ablett I and II) and 19 patients had severe/very severe tetanus (Ablett III and IV). Fifteen (50%) patients in ANS group required ventilation as compared to 28 (45%) in non-ANS group (p = 0.09). Fourteen (47%) patients died in ANS group as compared to 10 (15%) in non ANS group (p= 0.002). Out of those 14 patients died in ANS group, six patients had mild to moderate tetanus and eight patients had severe/ very severe tetanus. Major cause of death was cardiac arrhythmias (13/14; 93%) in ANS group and respiratory arrest (7/10; 70%) in non ANS group. Ten (33%) patients had complete recovery in ANS group while in non ANS group 35(48%) patients had complete recovery (p= 0.05).

**Conclusions:**

ANS dysfunction was present in one third of our tetanus population. 40% patients with ANS dysfunction had only mild to moderate tetanus. ANS dysfunction, irrespective of the need of mechanical ventilation or severity of tetanus, predicted poor outcome.

## Background

Tetanus is still a major health problem in developing world. Care of this potentially fatal, though preventable, disease has been revolutionized with advent of mechanical ventilation. However, still a significant minority, at least 10%, would die despite every effort [1]. Respiratory arrest used to be a common problem during preventilation era, however, ANS dysfunction has been emerged as a major problem in these patients in post ventilation era [1]. ANS dysfunction can cause a variety of arrhythmias which can potentially be lethal. There are reports that certain arrhythmias e.g. tachycardia carry poor prognosis [2], however, ANS dysfunction has been attributed to severe tetanus, which carry poor prognosis anyways. Violent autonomic disturbances, severe hypertension and tachycardia alternating with hypotension and bradycardia is reported in relation to severe tetanus. The association of so called milder forms i.e. stages I and II on Ablett's criteria [3] to ANS dysfunction and its affect on outcome is not well established. Our study highlights the importance of this previously not well-known aspect of tetanus.

## Methods

All patients admitted to two tertiary care centers in Karachi, Pakistan over a period of 10 years, were identified through ICD-9 coding system of the hospital medical records. The charts were reviewed retrospectively. The demographic, clinical, laboratory data was recorded and analyzed.

The autonomic dysfunction was defined as presence of labile or persistent hypertension (>140/90 mmHg) or hypotension (<90/60 mmHg) and persistent sinus tachycardia (heart rate >100 bpm), tachyarrhythmia or bradycardia (heart rate <50 bpm) alternating with tachycardia on EKG. These values were recorded at resting state, not during tetanic spasm. Brief episodes of tachycardia or hypertension are common in ICU and could be related to pain or anxiety. The values in ICU were mostly recorded in sedated and relaxed position. Continuous automated blood pressure and heart rate monitoring was done in all ICU patients. Blood pressure and heart rate were checked every four hour for the ward patients. The data regarding arterial lines is not available. The values recorded during tetanic spasms were not included in the study. The decision to admit these patients was based mostly on availability of ICU beds. Patients with only locked jaw without any autonomic abnormalities on admission were electively admitted to ward.

Severity of tetanus was classified based on Ablett criteria. The poor outcome was defined as either death or partial recovery at the time of discharge from the hospital. Partial recovery was defined as neck pain, back pain, walking difficulty and abnormal gait at discharge, in various combinations. Complete recovery was defined as absence of all of the symptoms and normal gait on examination at discharge.

The data was analyzed on SPSS version 10.0. Statistical analysis employed descriptive and univariate (chi-square and *t *test) methods.

## Results

Ninety six patients were identified. Sixty four (67%) were men and 32 (33%) were women. Their mean age was 44 (11–75) years. Identifiable risk factors were present in only 49 (51%) patients, recent trauma being most common which was noted in 27(28%) of the patients. Other risk factors were skin wounds, either infected or uninfected; 10 (11%), needle injury; 8 (9%) and recent surgery; 8 (9%).

Most common clinical features were trismus; 89 (93%), neck stiffness; 73 (76%), muscle spasms; (67) 70%, followed by dysphagia; 57 (59%), back stiffness; 42 (44%), dysarthria; 19 (20%), abdominal pain; 10 (11%) and subjective breathing difficulty; 7 (8%).

All patients received TIG (500–8000 international units) and almost all (99%) received benzodiazepine infusion. About one third (34%) of the patients received paralytics and 4 (5%) were also given magnesium sulphate. Majority of patients (71; 72%) were given antibiotics.

The EKG abnormalities include sinus tachycardia in 44 (46%) patients, cardiac ischemia in 5 (5%), sinus bradycardia in 2 (2%) and heart block in 1 (1%) patient. Blood pressure abnormalities include persistently elevated blood pressure in 8 (8%) patients, persistent hypotension in 2 (2%) and labile hypertension in 14 (15%).

Forty five (47%) patients recovered fully, 27 (28%) had partial recovery and 24 (25%) patients died. Major cause of death was cardiac arrhythmias (14/24; 58%) followed by respiratory arrest (7/24; 29%). All of the patients who had respiratory arrest were in general wards and never intubated. Fifty eight (60%) patients admitted to ICU. 43 (44%) required mechanical ventilation. Mean hospital stay was 17 (1–63) days. The hospital stay was prolonged in patients who required ventilation (25 vs 9 days)

ANS group had 31 (32%) patients while non ANS group comprised of 65 (68%) patients.

Severity of tetanus in ANS group was mild to moderate (Ablett I and II); 12 patients and severe/very severe (Ablett III and IV); 19 patients. 15 patients out of these 19 with severe tetanus required mechanical ventilation. Fourteen of 31 (47%) patients died in ANS group as compared to 10/62 (15%) in non ANS group (p= 0.002) (Table 1). Out of 14 patients who died, six had mild to moderate tetanus and eight patients had severe tetanus. Cause of death was cardiac arrhythmias (13 patients) and sepsis (1 patient) in ANS group while in non ANS group 7 patients died of respiratory arrest, one died of sepsis and one died of myocardial infarction. Cause of death was not known in one patient. None of these patients were witnessed to die during tetanic spasm. All patients who died were receiving benzodiazepines but not at a dose that could cause respiratory failure. All patients in ICU and majority of patients in the ward were receiving DVT prophylaxis in form of subcutaneous heparin and compression stockings. Ambulatory, non-bedridden patients (considered low risk for DVT) were not given DVT prophylaxis. Pulmonary embolism was not considered as a cause of death in any of the patients because these patients had no clinical evidence of DVT. The possibility that these patients died of acute autonomic dysfunction or arrhythmia could not be ruled out. Autopsy was not performed in any of the patients. Ten of 31 (33%) patients had complete recovery in ANS group as compared to 35/62 (48%) in non ANS group (p= 0.05). Both groups were similar in other aspects (Table 2)

**Table 2:** Tetanus severity, autonomic dysfunction and mortality.

Tetanus severity	ANS dysfunction (n = 31)	Non-ANS dysfunction (n = 65)
>Mild/Moderate	12 (6; died)	40 (1; died)
severe	19 (8; died)	25 (9; died)

**Table 1:** Comparison of Autonomic neuropathy versus no autonomic dysfunction in patients with tetanus.

Variable	ANS group (n = 31)	Non ANS group (n = 65)	P value
Mean age	39 years	46 years	0.2
Male: Female	23:8	41:24	0.1
Locked jaw	89 (93%)	89 (93%)	0.5
Dysphagia	18 (58%)	39 (60%)	0.12
Ventilation	15 (48%)	28 (43%)	0.09
Death	15 (48%)	10 (15%)	0.002
Complete recovery	10 (32%)	35 (54%)	0.05
Partial recovery	7 (23%)	20 (31%)	0.25

## Discussion

Tetanus is disease of antiquity. It was first described in Egypt over 3000 years ago [1]. It is caused by tetani clostridium which is an obligate non aerobe and ubiquitous organism.

Tetanus kills approximately one million people every year world wide and is a major health problem in developing world [1].

The clinical presentation of our patients is similar to that described in literature [2,4,5], most common presenting features being, trismus, neck stiffness and spasms (vide supra).

Prognosis of patients with tetanus has been variable. Overall mortality is approximately 10–50% [1,6], however, it is as high as 90–95% in certain select groups i.e. neonates [7] and tetanus associated with intramuscular quinine [8]. Various factors have been known to affect the prognosis. The poor prognostic factors include shorter incubation and onset periods [2,9], fever [9], tachycardia [2], fluctuating blood pressure [2], tetanus associated with intramuscular injections specially quinine [8], extreme of ages [1,7], hypoxia and acidosis at admission [10]. The major complication used to be respiratory arrest before advent of artificial ventilation, however, nosocomial infections and autonomic disturbances are major complications in post ventilator era [1]. Though the autonomic instability has been recognized as a major complication in the post ventilator era, this has been known since 1960s [11] and reported to occur in about a third of the tetanus patients [4]. An electrocardiographic study from India showed the incidence of sinus tachycardia as high as 85% [12]. The autonomic disturbance often develops a few days later in course. Pathogenesis of autonomic disturbances is unclear, however, several theories have been put forward, including damage to brain stem and hypothalamic nuclei [5] and direct disturbances in autonomic nerves [5,13]. The role of tetanus induced damage to brain stem nuclei has been discarded by many authorities. [14] Initially these were considered to be sympathetic overactivity [11], however, later studies with hemodynamic monitoring showed that both sympathetic as well as parasympathetic systems are involved [13]. The autonomic disturbances can be fatal. Though sudden cardiac arrest is most devastating complication, various tachy and bradyarrhthmias can be fatal. We also noted that the autonomic system dysfunction was associated with higher mortality (table I). In our cohort the major cause of death in patients with ANS dysfunction was cardiac arrhythmias. The plausible mechanisms for higher mortality in patients with ANS dysfunction include fatal tachyarrhthmias e.g. VF, cardiac arrest, myocardial infarction. Complications of intervention for ANS dysfunction is another potential mechanism of catastrophic end result as these patients are exquisitely sensitive to pharmacotherapy. To avoid these complication careful hemodynamic monitoring and cautious and judicious use of fluids and drugs like beta blockers, atropine may improve outcome in these patients. Morphine has been reported to control autonomic dysfunction. [15] It was used in only a few patients in our series in ICU patients, however, not as first line therapy. Data prior to use of morphine was used for presence or absence of autonomic dysfunction.

The ANS disturbances are considered to be part of severe tetanus [1], however, we did note the disturbances even in milder cases. As mentioned earlier the ANS dysfunction carries poor prognosis but this has been considered to be part of severe tetanus [1], which carries poor prognosis anyways. The association of so called milder forms i.e. stages I and II on Ablett's criteria [3] to ANS dysfunction and its affect on outcome is not well established. In our cohort 40% of the patients with ANS dysfunction were in grade I or II.

Our study is limited by its retrospective nature and paucity of hemodynamic and autonomic testing. However, based on the results we conclude that the presence of autonomic nervous dysfunction irrespective of the respiratory/ventilatory status, predicts poor outcome in tetanus. Studies have shown that reduced tetanus mortality in the recent era is most probably due to advances in ICU management. [16] We also strongly think that the ANS dysfunction is inherent to tetanus even in milder cases so all patients with tetanus should be carefully monitored in ICU settings to avoid mortality from this deadly but potentially reversible disease. Further prospective studies are required to confirm our findings

## Conclusions

Autonomic dysfunction is not uncommon in patients with Tetanus. It was present in one third of our tetanus population. 40% patients with ANS dysfunction had only mild to moderate tetanus. ANS dysfunction, irrespective of the need of mechanical ventilation or severity of tetanus, predicted poor outcome.

## Competing interests

The author(s) declare that they have no competing interests.

## Authors' contributions

MW: conceptualization, data analysis, manuscript preparation. BAK: data entry and analysis, statistical analysis, manuscript preparation. NT: data acquisition, manuscript preparation. RS: data acquisition, data analysis, manuscript preparation. NAS: conceptualization, manuscript preparation. NS: conceptualization, manuscript preparation.

## Pre-publication history

The pre-publication history for this paper can be accessed here:


